# Supplementary material for: Polyketide synthase gene clusters in the ericoid mycorrhizal fungus Oidiodendron maius: genome-wide prediction, expression profiling, and functional characterization of knockout mutants
Source: Front Plant Sci. 2026 Sep 16;17:1924430. doi: 10.3389/fpls.2026.1924430 (PMC13623903; doi:10.3389/fpls.2026.1924430)
Supplement: Supplementary file 1 [file SupplementaryFile1.zip › Supplementary Materials/Supplementary Folder 1.zip!/Oidma1_AssemblyScaffolds_Repeatmasked/knownclusterblast/cluster52/jgi.p_Oidma1_173296_mibig_hits.html]

| MIBiG Protein | Description | MIBiG Cluster | MiBiG Product | % ID | % Coverage | BLAST Score | E-value |
| --- | --- | --- | --- | --- | --- | --- | --- |
| AGO59046.1 | PtaM | BGC0000121 | Pestheic\_acid\_biosynthetic\_gene\_cluster | 71 | 95.2029520295 | 757 | 8.5e-219 |
| EAQ86388.1 | hypothetical\_protein | BGC0001405 | Chaetoviridin\_/\_chaetomugilin\_biosynthetic\_gene\_cluster | 53 | 97.4169741697 | 522 | 6e-148 |
| BAE56588.1 |  | BGC0001123 | Aspirochlorine\_biosynthetic\_gene\_cluster | 51 | 96.6789667897 | 522 | 7.8e-148 |
| ADI24948.1 | GsfI | BGC0000070 | Griseofulvin\_biosynthetic\_gene\_cluster | 52 | 96.1254612546 | 512 | 6.2e-145 |
| ACD39771.1 | non-heme\_halogenase | BGC0000134 | Radicicol\_biosynthetic\_gene\_cluster | 56 | 81.5498154982 | 497 | 2.7e-140 |
| ALT31850.1 | ArmH3 | BGC0001401\_c3 | Melleolides\_biosynthetic\_gene\_cluster | 45 | 96.4944649446 | 423 | 3.8e-118 |
| AEM76786.1 | putative\_halogenase | BGC0001401\_c2 | Melleolides\_biosynthetic\_gene\_cluster | 44 | 98.3394833948 | 416 | 6e-116 |
| AEM76785.1 | putative\_halogenase | BGC0001401 | Melleolides\_biosynthetic\_gene\_cluster | 43 | 98.5239852399 | 408 | 1.6e-113 |
| ALT31852.1 | ArmH5 | BGC0001401\_c5 | Melleolides\_biosynthetic\_gene\_cluster | 42 | 97.0479704797 | 393 | 4.2e-109 |
| ALT31851.1 | ArmH4 | BGC0001401\_c4 | Melleolides\_biosynthetic\_gene\_cluster | 44 | 99.815498155 | 388 | 1.4e-107 |
| ADE22292.1 | putative\_non-heme\_halogenase | BGC0000279 | Xantholipin\_biosynthetic\_gene\_cluster | 38 | 79.520295203 | 278 | 1.5e-74 |
| ABM21576.1 | CrpH | BGC0000975 | Cryptophycin\_biosynthetic\_gene\_cluster | 35 | 85.9778597786 | 278 | 1.5e-74 |
| AMK51270.1 | Hex13 | BGC0001376 | Hexaricin\_biosynthetic\_gene\_cluster | 37 | 79.3357933579 | 268 | 2e-71 |
| CAM34371.1 | putative\_non-heme\_halogenase | BGC0000242 | Lysolipin\_biosynthetic\_gene\_cluster | 37 | 76.9372693727 | 262 | 1.1e-69 |
| CAJ46693.1 | tryptophan\_halogenase | BGC0000969 | Chondramide\_biosynthetic\_gene\_cluster | 31 | 100.184501845 | 246 | 6.3e-65 |
| ACU71639.1 | tryptophan\_halogenase | BGC0001154 | Cacibiocin\_biosynthetic\_gene\_cluster | 31 | 84.8708487085 | 225 | 1.2e-58 |
| AEU17894.1 | putative\_halogenase | BGC0001072 | Simocyclinone\_biosynthetic\_gene\_cluster | 31 | 76.9372693727 | 222 | 1.3e-57 |
| WP\_052390547.1 | halogenase | BGC0001308 | Simocyclinone\_D9\_/\_simocyclinone\_D10\_/\_simocyclinone\_D11 | 31 | 76.7527675277 | 222 | 1.7e-57 |
| ADU85999.1 | putative\_tryptophan\_halogenase | BGC0000165 | Tiacumicin\_B\_biosynthetic\_gene\_cluster | 30 | 76.1992619926 | 203 | 4.7e-52 |
| AAN65237.1 | putative\_halogenase | BGC0000832 | Clorobiocin\_biosynthetic\_gene\_cluster | 33 | 64.2066420664 | 183 | 6.6e-46 |
| AAL06656.1 | alkylhalidase | BGC0000965 | C-1027\_biosynthetic\_gene\_cluster | 30 | 80.258302583 | 177 | 4.7e-44 |
| ALU98528.1 | tryptophan\_halogenase | BGC0001397 | Lidamycin\_biosynthetic\_gene\_cluster | 30 | 78.7822878229 | 176 | 6.2e-44 |
| CCA54215.1 | FAD-binding\_protein | BGC0000893 | Chloramphenicol\_biosynthetic\_gene\_cluster | 31 | 69.1881918819 | 176 | 8.1e-44 |
| AGC09515.1 | FAD\_dependent\_halogenase | BGC0001183 | Lobophorin\_biosynthetic\_gene\_cluster | 32 | 69.557195572 | 171 | 2e-42 |
| AAK81830.1 | non-heme\_halogenase | BGC0000326 | Complestatin\_biosynthetic\_gene\_cluster | 33 | 66.7896678967 | 171 | 3.4e-42 |
| AEI58871.1 | halogenase | BGC0000455 | Vancomycin\_biosynthetic\_gene\_cluster | 29 | 81.5498154982 | 169 | 9.9e-42 |
| AGS77325.1 | Halogenase | BGC0001178 | UK-68,597\_biosynthetic\_gene\_cluster | 32 | 66.4206642066 | 168 | 1.7e-41 |
| CAE53362.1 | halogenase | BGC0000440 | Teicoplanin\_biosynthetic\_gene\_cluster | 29 | 80.8118081181 | 166 | 1.1e-40 |
| CAG15020.1 | halogenase | BGC0000441 | Teicoplanin\_biosynthetic\_gene\_cluster | 29 | 80.8118081181 | 166 | 1.1e-40 |
| AFV52210.1 | aryl-ACP\_halogenase | BGC0000081 | Kedarcidin\_biosynthetic\_gene\_cluster | 30 | 86.3468634686 | 165 | 1.4e-40 |
| AAM80532.1 | StaI | BGC0000290 | A47934\_biosynthetic\_gene\_cluster | 28 | 91.1439114391 | 165 | 1.9e-40 |
| ctg1\_orf12 |  | BGC0001366 | BE-24566B\_/\_zunyimycin\_A\_biosynthetic\_gene\_cluster | 32 | 70.110701107 | 165 | 1.9e-40 |
| CAA76550.1 | halogenase | BGC0000311 | Balhimycin\_biosynthetic\_gene\_cluster | 29 | 81.5498154982 | 165 | 2.4e-40 |
| ADM46362.1 | halogenase | BGC0000106 | Naphthomycin\_biosynthetic\_gene\_cluster | 30 | 70.110701107 | 164 | 4.1e-40 |
| CAA11780.1 | PCZA361.26 | BGC0000322\_c2 | Chloroeremomycin\_biosynthetic\_gene\_cluster | 29 | 81.5498154982 | 163 | 5.4e-40 |
| ABS50488.1 | NapH2 | BGC0000652 | Napyradiomycin\_biosynthetic\_gene\_cluster | 32 | 61.2546125461 | 161 | 3.5e-39 |
| ABS50460.1 | NapH2 | BGC0001079 | Napyradiomycin\_biosynthetic\_gene\_cluster | 32 | 61.2546125461 | 161 | 3.5e-39 |
| ABD65950.1 | halogenase | BGC0000341 | Enduracidin\_biosynthetic\_gene\_cluster | 29 | 86.1623616236 | 160 | 7.8e-39 |
| CAD91205.1 | halogenase | BGC0000289 | A40926\_biosynthetic\_gene\_cluster | 31 | 66.4206642066 | 158 | 1.7e-38 |
| ACE80255.1 | HrmQ | BGC0000374 | Hormaomycin\_biosynthetic\_gene\_cluster | 32 | 66.0516605166 | 158 | 2.3e-38 |
| ABY66006.1 | halogenase | BGC0001008 | Maduropeptin\_biosynthetic\_gene\_cluster | 31 | 71.9557195572 | 158 | 2.3e-38 |
| AAM54090.1 | halogenase | BGC0000020 | Ansamitocin\_biosynthetic\_gene\_cluster | 31 | 69.557195572 | 157 | 3.9e-38 |
| AGS77327.1 | Halogenase | BGC0001178 | UK-68,597\_biosynthetic\_gene\_cluster | 29 | 66.4206642066 | 141 | 2.9e-33 |
| AAM70353.1 | CalO3 | BGC0000033 | Calicheamicin\_biosynthetic\_gene\_cluster | 29 | 68.2656826568 | 141 | 2.9e-33 |
| ESP90845.1 | Dehydrogenases\_(flavoprotein) | BGC0000891 | Bromophenols\_/\_bromopyrroles\_biosynthetic\_gene\_cluster | 30 | 63.6531365314 | 139 | 1.1e-32 |
| AHA34035.1 | Bmp2 | BGC0000890 | Bromophenols\_/\_bromopyrroles\_biosynthetic\_gene\_cluster | 29 | 63.6531365314 | 137 | 5.4e-32 |
| AAM80530.1 | StaK | BGC0000290 | A47934\_biosynthetic\_gene\_cluster | 28 | 66.6051660517 | 134 | 4.6e-31 |
| ABQ04143.1 | tryptophan\_halogenase | BGC0000838 | Flexirubin\_biosynthetic\_gene\_cluster | 25 | 77.4907749077 | 111 | 3.2e-24 |
